# On Scarlatina and Its Successful Treatment by the Acidum Aceticum Dilutum of the Pharmacopœia

**Published:** 1846-04

**Authors:** 


Art. V.—*On Scarlatina and its Successful Treatment by the Acidum Aceticum Dilutum of its Pharmacopœia.* By Isaac B. Brown.—*London,* 1846. 8vo, pp. 66.

This pretty little book is like the Dead Sea apples—fair without, but naught within. It seems as if it came forth on purpose to illustrate some of the statements in the article "Homœopathy, Allopathy, &c.," in our last number. The good man who writes it, no doubt, conscientiously believes that the cases of scarlatina treated by him, would have all gone wrong but for his "sheet-anchor, acetic acid," and his "other remedies as auxiliaries;" and equally conscientiously, no doubt, did the famous fly in the fable glorify itself for the successful revolution of the wheel whereon it sat. Poor Nature, what wouldst thou have done, without the help of our good doctor? Thy wheel would assuredly have stood still under the pressure of the scarlet plague, but for the oiling of the axle with his acid. And should we marvel thereat, knowing, as we know from the lore of Isaac B. Brown, that, in this wondrous acid, this nepenthe rare, "we have all we want, for destroying the poison of the blood, for allaying the vascular state of the mucous membrane, for preventing hemorrhage from the blood-vessels, astringing the tonsils and fauces, and for checking the febrile action and supplying fresh chyle to the blood." (p. 10.) And yet we marvel at one thing, O Isaac B. Brown, and this is—that with such an all-sufficient, and more than all-sufficient, panacea in thy hand, thou shouldst have needed any auxiliaries, and least of all such auxiliaries as thou soughtest and usedst. Most firmly do we believe that if thou hadst satisfied thyself with the pleasant potions of acidulated syrup and water, thy patients would have all got well quite as fast, and blessed thee for saving them from thy naughty and nasty auxiliaries.

But the leaven of the old man of drugs was too strong in good Isaac Brown, to allow him to be content with the really excellent treatment of the acidulated ptisans,* recommended to him by the "assistant to Mr. George Yates Hunter, of Margate," and thus, therefore, does he torment his poor patients *secundum artem:*—They are carefully purged by calomel, castor oil, rhubarb, and magnesia, their tonsils sponged with a solution of nitrate of silver, their throats rubbed with soap and camphor liniments and laudanum, or surrounded "from ear to ear," with linseed poultices kept constantly applied, their bodies affused with vinegar, &c., the room sprinkled with a solution of chloride of lime, and last, but not least, the patient's strength supported by bark, ether, mutton-broth, brandy, port wine, &c.

How does the author reconcile the two following statements, in pages viii and ix.? In the first he tells us, his treatise is intended for "parents and heads of families," as well as for the "members of the medical profession;" in the next, that there is the utmost danger in any one not "qualified to practise the medical science," "to carry out this or any other plan, *without first obtaining the best advice the locality in which they reside affords.*" Is this, like the rest of his treatise, a bit of *post-hoc-ergo-propter-hoc* reasoning, which he wishes to diffuse among the philosophic fathers and mothers of his "locality?"

* "Diluted distilled vinegar, two drachms; syrup, four drachms; distilled water, two ounces. Mix and take a fourth part every three hours." We would suggest as a great improvement in this formula to quadruple the proportion of water, and then double or yet further increase the amount of the dose!

